# Attenuated Induction of the Unfolded Protein Response in Adult Human Primary Astrocytes in Response to Recurrent Low Glucose

**DOI:** 10.3389/fendo.2021.671724

**Published:** 2021-05-26

**Authors:** Paul G. Weightman Potter, Sam J. Washer, Aaron R. Jeffries, Janet E. Holley, Nick J. Gutowski, Emma L. Dempster, Craig Beall

**Affiliations:** ^1^ Institute of Biomedical and Clinical Sciences, University of Exeter Medical School, Exeter, United Kingdom; ^2^ Royal Devon and Exeter Hospital, University of Exeter Medical School and the Department of Neurology, Exeter, United Kingdom

**Keywords:** recurrent low glucose, unfolded protein response, ER stress, human primary astrocytes, transcriptome (RNA-seq)

## Abstract

**Aims/hypothesis:**

Recurrent hypoglycaemia (RH) is a major side-effect of intensive insulin therapy for people with diabetes. Changes in hypoglycaemia sensing by the brain contribute to the development of impaired counterregulatory responses to and awareness of hypoglycaemia. Little is known about the intrinsic changes in human astrocytes in response to acute and recurrent low glucose (RLG) exposure.

**Methods:**

Human primary astrocytes (HPA) were exposed to zero, one, three or four bouts of low glucose (0.1 mmol/l) for three hours per day for four days to mimic RH. On the fourth day, DNA and RNA were collected. Differential gene expression and ontology analyses were performed using DESeq2 and GOseq, respectively. DNA methylation was assessed using the Infinium MethylationEPIC BeadChip platform.

**Results:**

24 differentially expressed genes (DEGs) were detected (after correction for multiple comparisons). One bout of low glucose exposure had the largest effect on gene expression. Pathway analyses revealed that endoplasmic-reticulum (ER) stress-related genes such as *HSPA5*, *XBP1*, and *MANF*, involved in the unfolded protein response (UPR), were all significantly increased following low glucose (LG) exposure, which was diminished following RLG. There was little correlation between differentially methylated positions and changes in gene expression yet the number of bouts of LG exposure produced distinct methylation signatures.

**Conclusions/interpretation:**

These data suggest that exposure of human astrocytes to transient LG triggers activation of genes involved in the UPR linked to endoplasmic reticulum (ER) stress. Following RLG, the activation of UPR related genes was diminished, suggesting attenuated ER stress. This may be a consequence of a successful metabolic adaptation, as previously reported, that better preserves intracellular energy levels and a reduced necessity for the UPR.

## Introduction

Iatrogenic hypoglycaemia is a limiting factor to optimal glycaemic control in people with type 1 (T1D) and insulin/sulphonylurea-treated type 2 diabetes [T2D (1)]. Acutely, severe hypoglycaemia, defined as requiring help from a third party for recovery, can lead to brain damage or death, in extreme but rare circumstances. Importantly the detection of hypoglycaemia and activation of appropriate counterregulatory responses (CRR) to reverse hypoglycaemia, are mediated to large extent by the central detection of hypoglycaemia (2). Moreover, frequent exposure to hypoglycaemia leads to defective CRR. Specifically the magnitude of the glucose-raising catecholamine response during hypoglycaemia is suppressed and triggered at a lower plasma glucose level, combined with an often absent glucagon response (3). These changes are, at least in part, driven by changes in brain hypoglycaemia-sensing nuclei, including the ventromedial hypothalamus [VMH (4)] and hindbrain (5).

Activation of CRR can be induced by administration of glucoprivic agents such as 2-deoxy-glucose [2DG (6)] and 5-thio-D-glucose (7) to discrete brain nuclei. Glucose sensing neurons found in nuclei of the hypothalamus and hindbrain detect changes in glucose concentration (8, 9). However, recently astrocytes have been implicated in direct glucose sensing and altering neuronal output (10, 11). For example, the expression of the glucose transporter GLUT2 is required in astrocytes but not neurons for a robust response to glucoprivation (12). Moreover, astrocytes in *ex vivo* brain slices containing the nucleus of the tractus solaris [NTS (6)], were activated by low glucose or 2DG. Furthermore, blocking astrocytic metabolism with fluorocitrate prevented increases in gastric motility normally associated with hypoglycaemia (13). In response to low glucose, astrocytes in the NTS increase intracellular calcium levels which occur before and independently of neuronal activity (14). Recently it has also been shown that blockade of purinergic signalling from astrocytes also blocks 2DG-induced CRR (11, 15). In addition, astrocytic glutamate uptake is impaired following RH, contributing to counterregulatory failure (16). Together these data suggest an active role of astrocytes in glucose detection, despite this evidence little is known about the intrinsic changes within astrocytes, especially human astrocytes, following recurrent low glucose (RLG). In this study, we used both RNA sequencing and an epigenome-wide association study (EWAS) of DNA methylation (DNAm) to examine for the first time, changes to the human astrocyte transciptome and methylome following acute and recurrent low glucose exposure.

## Research Design and Methods

### Astrocyte Isolation and Cell Culture

**Figure 1:**
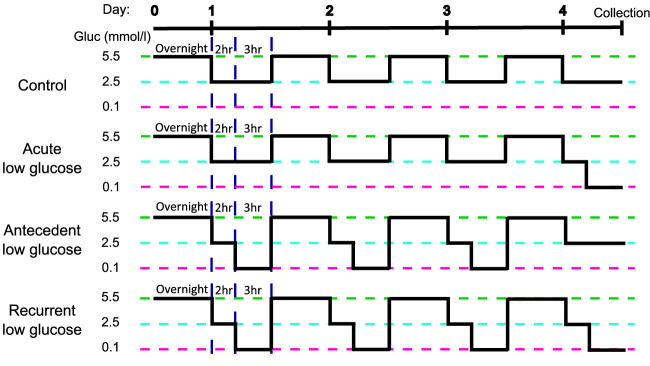
Schematic of the recurrent low glucose model. Human primary astrocytes were exposed to 0, 1, 3, or 4, three-hour long bouts of 0.1 mmol/l glucose; control (C), acute low glucose (LG), antecedent recurrent low glucose (aRLG), and recurrent low glucose (RLG) respectively. Each day cells were first incubated in 2.5 mmol/l glucose for 2 hours as a step down from overnight/stock media of 5.5 mmol/l glucose. Adapted from (18).

HPA cells were isolated from post-mortem sub-ventricular deep white matter following consent from next-of-kin, and with ethical approval from the North and East Devon Research Ethics Committee and confirmed as glial fibrillary and acidic protein (GFAP) and vimentin positive, as previously described (17), confirming astrocyte identity. The recurrent low glucose (RLG) model has been previously described [(18); **Figure 1**]. Each day cells were cultured in 2.5 mmol/L glucose-containing media for 2 hours before being changed for media containing 0.1 (low) or 2.5 (normal) mmol/L glucose for 3 hours. Overnight, cells were recovered in stock media containing 5.5 mmol/L glucose. This was repeated for four days. Control and low glucose (LG) treated cells had 2.5 mmol/L glucose for three days and on the fourth day the LG group received low glucose for 3 hours. The antecedent RLG (aRLG) and RLG groups had 0.1 mmol/L glucose for 3 hours on three consecutive days, on the fourth day the aRLG group was exposed to 2.5 mmol/L glucose, whereas RLG was exposed to 0.1 mmol/L glucose for 3 hours. Mannitol was added to maintain osmolarity (see ESM for details). Samples were split for RNA extraction and DNA extraction, with a total of five and six replicates for RNA sequencing and DNA methylation studies, respectively. Cells were confirmed as mycoplasma free using the MycoAlert kit (Lonza, Slough, UK).

### RNA Sequencing

Briefly, RNA was extracted using TRIzol and Direct-zol miniprep kit (Invitrogen, Carlsbad, CA, USA), according to manufacturers’ instructions. cDNA libraries were generated using the TruSeq DNA HT Library Preparation Kit (Illumina Inc., San Diego, CA, USA). Sequencing reads were generated using the Illumina HiSeq 2500 and fastq sequence quality was checked using MultiQC before alignment to the human genome (Build GRCh38.p12) using STAR. Mapped reads were counted using the FeatureCounts function of the subread package. Differential gene expression was calculated using DESeq2 (19) using the Likelihood ratio test function to analyse all groups together followed by the Wald-test for pairwise analysis. Genes with a false discovery rate (FDR) ≤0.05 were considered differentially expressed. For a principal component analysis plot see ESM **Figure 1**. Functional gene ontology analysis was performed using GOSeq. Gene length was accounted for during GO analysis. Raw RNAseq files are available through GEO accession number GSE166848.

### DNA Methylation Analysis

DNA was extracted using a modified phenol:chloroform protocol and DNA methylation (DNAm) examined using the Infinium MethylationEPIC BeadChip platform (Illumina Inc.; EPIC). 729727 probes remained after QC processes. The one-way analysis of variance (ANOVA) test was used to test for differentially methylated sites associated across the three groups: LG, aRLG, RLG compared to control. To determine which group was driving the association behind the significant ANOVA results, the *T* statistics for control versus each of the three groups were extracted from the regression model. Unprocessed array data is available through GEO accession number GSE166848.

## Results

### Low Glucose-Induced Changes in Gene Expression in Human Astrocytes

**Figure 2:**
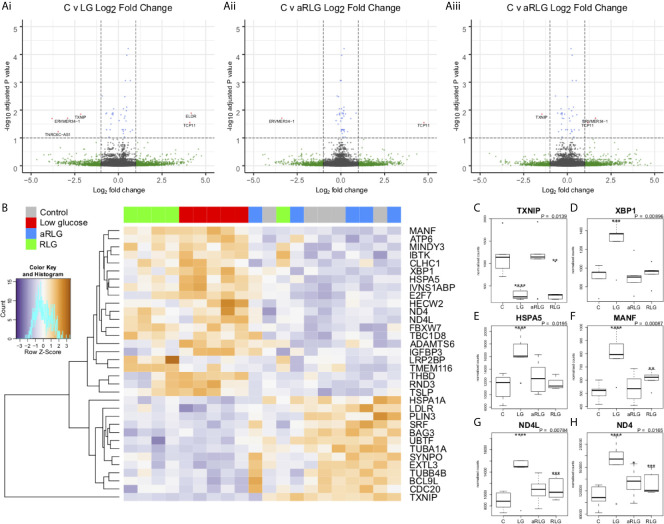
Glucose variation alters expression of genes involved in endoplasmic-reticulum stress. Volcano plots on the pairwise differential expression analysis between control cells (C) versus (Ai) low glucose (LG), (Aii) antecedent RLG, and (Aiii) recurrent low glucose (RLG), the red points on the plots represent genes padj<0.05. **(B)** Heatmap of hierarchical clustering of LRT analysis FDR ≤ 0.1 indicates differentially expressed genes (rows) between the four groups (padj<0.1). Orange indicates up-regulation and blue indicates down-regulation. The LG and RLG groups cluster together. *TXNIP* **(C)**, *XBP1* **(D)**, *HSPA5* **(E)**, *MANF* **(F)**, *ND4L* **(G)**, *ND4* **(H)** expression profiles, selected for their functional relevance to hypoglycaemia (*p*-value is the adjusted result of the likelihood ratio test). n=5; *p<0.05, **p<0.01, ***p<0.001, ****p<0.0001. Data presented as Mean ± SD.

In HPA cells, expression of 1240 genes were significantly (*p*<0.05) altered in response to glucose variation; 24 of which were significantly differentially expressed (DE) after FDR correction (adjusted *p*<0.05; **Figure 2A**). Volcano plots displaying the pairwise comparisons of each treatment group versus control shows that LG (**Figure 2Ai**) produced the largest effect on gene expression, whereas changes induced by aRLG (**Figure 2Aii**) and RLG (**Figure 2Aiii**) were more modest. LG and RLG shared similar DE patterns (**Figure 2B**) and importantly *TXNIP*, regulated by glucose (20), was significantly downregulated in both LG (log2 fold-change -2.16, *p*=1.09E-5) and RLG (log2 fold-change -1.46, *p*=2.91E-3; **Figure 2C**). Of the other DE genes there was a predominance of genes related to endoplasmic reticulum (ER)-stress. X-box binding protein 1 (*XBP1*; log2 fold-change 0.28, *p*=1.56E-4; **Figure 2D**), heat shock protein family A member 5 (*HSPA5*; log2 fold-change 0.34, *p*=3.55E-6; **Figure 2E**), and mesencephalic astrocyte-derived neurotrophic factor (*MANF*; log2 fold-change 0.41, *p*=7.55E-6; **Figure 2F**) showed increased expression following LG exposure which was blunted following RLG. Similarly, mitochondrially encoded NADH:ubiquinone oxidoreductase core, subunit 4 and subunit 4L (*ND4* and *ND4L*) had increased gene expression in acute LG (*ND4*; log2 fold-change 0.37, *p*=3.5E-6; *ND4L*; log2 fold-change 0.47, *p*=5.75E-7) and a diminished, but still significant increase following RLG (**Figures 2G, H**). Pathway analysis of the DE genes identified seven gene ontology (GO) terms that were significantly altered after correction for multiple comparisons, which were related to the unfolded protein response (UPR) and ER-stress (**Table 1**).

**Table 1:** Glucose variation significantly enriched gene ontologies related to endoplasmic-reticulum stress.

GO term ID	GO term full name	Number of DEGs in the category	Total number of genes in the category	corrected *p* value
GO:0006986	response to unfolded protein	8	160	0.0159
GO:1905897	regulation of response to endoplasmic reticulum stress	6	72	0.0159
GO:0006984	ER-nucleus signalling pathway	5	45	0.0159
GO:0035966	response to topologically incorrect protein	8	179	0.0159
GO:0034620	cellular response to unfolded protein	7	125	0.0159
GO:0035967	cellular response to topologically incorrect protein	7	143	0.0329
GO:0036498	IRE1-mediated unfolded protein response	5	59	0.0440

Gene ontologies that were significantly enriched by the differentially expressed genes. All seven of the GO terms were related to endoplasmic-reticulum stress and the unfolded protein response.

### LG and RLG Produce Distinct DNA Methylation Profiles

**Figure 3:**
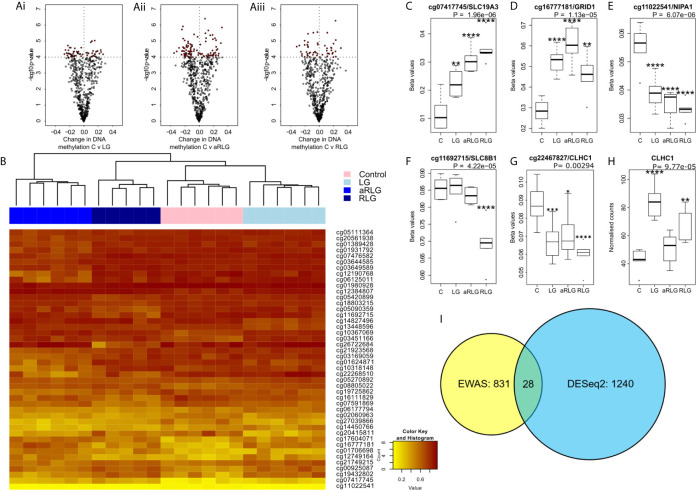
Effect of glucose variation on DNA methylation. **(A)** The most differentially methylated genes (ANOVA p ≤ 0.001) in pairwise comparison (red points are *p*<0.0001) between control treated HPA cells (C) versus (Ai) low glucose (LG), (Aii) antecedent recurrent low glucose (aRLG), and (Aiii) recurrent low glucose (RLG). **(B)** Heatmap of hierarchical clustering using probes ANOVA *p*<0.001 indicates differentially methylated cg sites (rows) between the four groups. Orange indicates hypermethylation and yellow indicates hypomethylation. Box plots of some of the most differentially methylated CpG sites labelled by their associated gene, selected for functional importance **(C)**, cg07417745/SLC19A3, **(D)**, cg16777181/GRID1, **(E)**, cg11022541/NIPA1, **(F)**, cg11692715/SLC8B1 **(G)**, cg22467827/CLHC1 (*p*-value is the adjusted result of the ANOVA). **(H)**, *CLCH1* gene expression increases. Error bars represent standard deviation **(I)**, Venn diagram of differentially methylated cg sites in yellow and differentially expressed genes (blue) and overlap between the two data sets, 28 genes. Data presented as Mean ± SD. *p<0.05, **p<0.01, ***p<0.001, ****p<0.0001. n=6 for methylation data and n=5 for gene expression changes.

Our analyses did not identify any differential methylated positions (DMP) that reached genome-wide significance for DNA methylation association analyses (**Figures 3Ai-iii**; *p*<9.42x10^-8^ (21)). However, 65 probes reached nominal significance of *p*<0.0001. Hierarchical clustering of these top probes showed four distinct groups that matched with the four experimental conditions suggesting a DNA methylation profile specific to each condition (**Figure 3B**). Of the differentially methylated CpG sites, several were related to energy or ion homeostasis. *SLC19A3* (cg07417745, *p*=5.16E-7, βΔ= 0.23), encoding the thiamine transporter was hypermethylated after LG showing a linear relationship with the number of bouts of LG exposure (**Figure 3C**). Similarly, methylation of the *GRID1* gene, encoding the ionotropic glutamate receptor δ1 (cg16777181) was hypermethylated following LG exposure (*p*=1.90E-3, βΔ=0.18) and this remained elevated following RLG (**Figure 3D**). In contrast, cg1102254 (*NIPA1*, *p*=2.65E-6, βΔ= -0.02), cg11692715 (*SLC8B1*; *p*=1.61E-5, βΔ= -0.16) and cg22467827 (*CLHC1*, *p*=4.28E-4, βΔ= -0.03), which encode a Mg^2+^ transporter (22), a Na^+^/Ca^2+^ antiporter, and clathrin heavy chain linker domain containing 1 respectively, were hypomethylated following RLG (**Figures 3E–G**). The probe cg22467827 (annotated to the gene *CLHC1*) was also differentially expressed (log2 fold-change 0.80, *p*=1.03E-4) in relation to RLG (**Figure 3H**). The two datasets (RNAseq and EPIC) were integrated resulting in 28 DE genes that overlapped with 31 differentially methylated positions (**Figure 3I**).

## Discussion

The central adaptations in response to RH that mediates defective CRR require further investigation, with little known about how astrocytes respond or adapt to RH. We sought to examine changes in HPA gene expression and DNA methylation to determine which, if any, pathways were altered by acute and RLG exposure. DE and GO pathway analyses revealed that the major pathway altered by acute LG was the UPR. Protein folding within the ER requires hydrolysis of ATP (for review see (23)) and reductions in ATP content driven by energy stress increases protein misfolding to activate the UPR (24). ER stress, *via* ATF6 promotes the production of XBP1 (25), which is spliced by IRE1α, to produce a potent transcriptional activator, XBP1s that increases HSPA5 (25) and MANF expression (26)). *MANF* is upregulated by UPR to inhibit cell proliferation and prevent ER-stress-related cell death (27, 28). Interestingly, here expression of *XBP1*, *HSPA5*, and *MANF* were increased following a single bout of LG. Similar ER stress responses have been reported in pericytes (29), cardiac tissue (30), rat primary astrocytes (31) and primary hippocampal neurons (24) in response to LG. Following RLG, the increase in UPR-related gene expression was substantially diminished. Given that energy deficiency increases ER stress, it is plausible that acute LG exposure causes poor folding of proteins leading to a marked increase in ER stress. Following successive bouts of LG, a concomitant metabolic adaptation, as previously reported (18), better preserves cellular (or intra-ER) ATP levels, thus attenuating (or delaying) subsequent LG-induced ER stress, reducing the necessity of the UPR. This is supported by the observation that expression levels of *ND4L* and *ND4* following RLG remained elevated above control. These mitochondrial genes encode two subunits of complex I (NADH dehydrogenase) and the continued elevation of expression following RLG suggests a persistent adaptation. This correlates with our previous data in the same cell type demonstrating increased basal mitochondrial oxygen consumption following RLG, mediated by an increased reliance on fatty acid oxidation for ATP generation (18). It is worth noting that in our previous study, we did not observe any reduction in total intracellular ATP content following acute or recurrent low glucose exposure. When combined with our data presented here, it is possible that normal ER functions are transiently reduced during acute low glucose exposure in order to maintain intracellular ATP levels. Whether any metabolic adaptation following RLG leads to better preservation of intra-ER ATP levels remains to be determined.

The EWAS identified 65 DMPs associated with LG/RLG that reached nominal significance, while we did not identify any DMPs that reached the suggested array-wide significance (*p*<9.42x10^-8^). Hierarchical clustering revealed distinct patterns of DNA methylation across the four conditions. One of the most significant DMPs (cg07417745) is located in intron 1 of the *SLC19A3* gene, which encodes a thiamine transporter (32), and showed a linear relationship between increased DNA methylation and the number of LG exposures. Interestingly, expression of this gene has previously been found to be modulated by hyperglycaemic-like conditions (33). Conversely, methylation of cg11022541 and cg11692715 located within the genes *NIPA1* and *SLC8B1* respectively, decreased following RLG. As these genes encode a Mg^2+^ transporter (22) and a Na^+^/Ca^+^ exchanger (34), this may indicate the energetic cost of ion handling within the cell, which requires further investigation. The main limitation in this study was that we were underpowered in the DNA methylation analyses as power analysis indicated we had 50% power to detect a difference of 10% in half of all the sites on the EPIC array. Moreover, the relationship between DNA methylation and gene expression is complex, with the direction of effect dictated by sequence context (35). Furthermore, the annotation of DNAm sites to genes is purely based on proximity rather than empirically derived data (36), both of these factors make inferences between DMPs and gene expression complicated. Despite these challenges we looked for overlapping genes between the datasets and identified 28 that were significantly altered (p<0.05) in both analyses. For example, *CLHC1* gene expression was significantly increased and a DMP (cg22467827) located in intron 1 was hypomethylated. This tentatively suggests that DNA methylation within the first intron may be mediating the upregulation of this gene in the response to LG glucose.

These data demonstrate the intrinsic response of adult human primary astrocytes to acute and recurrent low glucose exposure. Despite the advantages of the high resolution information obtained from primary astrocyte cultures, whether these responses are shared by astrocytes across different brain regions remains unknown, especially given the emerging evidence of astrocyte heterogeneity. In addition, the influence of neighbouring cells such as neurons, pericytes and microglia would be interesting to examine. Therefore, expanding these findings to a more replete setting will be important for future studies using for example human inducible pluripotent stem cells *in vitro* or *ex vivo*/*in vivo* rodent models.

In summary, there are both shared and unique gene expression and DNA methylation profiles in human astrocytes following LG and RLG exposure. A single bout of LG exposure induced expression of genes associated with the UPR linked to ER stress. This response diminished after four bouts of LG exposure, suggesting an attenuated stress response. Taken together with previous observations that astrocytes adapt to RLG by increasing reliance on fatty acid oxidation to maintain intracellular ATP levels, activation of the UPR by glucose deprivation may be attenuated following RLG exposure.

## Data Availability Statement

The datasets presented in this study can be found in online repositories. The names of the repository/repositories and accession number(s) can be found in the article/**Supplementary Material**.

## Author Contributions

AJ, ED, and CB conceived the project. PW, SW, AJ, and ED contributed to data acquisition, analyses and writing of the manuscript. JH and NG obtained human post mortem tissue from which astrocytes were isolated, cultured, and characterised as the stable human primary astrocyte population, thus permitting the investigation of intrinsic changes in human astrocyte responses to be undertaken. CB wrote and edited the manuscript and accepts full responsibility for the work and/or conduct of the study, had access to the data and controlled the decision to publish. All authors contributed to the article and approved the submitted version.

## Funding

This work was funded by a Novo Nordisk UK Research Foundation grant to CB, AJ, and ED, Mary Kinross Charitable Trust PhD studentship to CB for PWP, a European Foundation for the Study of Diabetes/Novo Nordisk Programme for Diabetes Research in Europe, a Diabetes UK RD Lawrence Fellowship to CB (13/0004647) and a JDRF postdoctoral fellowship (3-PDF-2020-941-A-N) to PWP. The University of Exeter Sequencing service is funded by Medical Research Council Clinical Infrastructure award (MR/M008924/1), Wellcome Trust Institutional Strategic Support Fund (WT097835MF), Wellcome Trust Multi-User Equipment Award (WT101650MA), and BBSRC LOLA award (BB/K003240/1). This study represents independent research supported by the National Institute of Health Research Exeter Clinical Research facility. The views expressed are those of the author(s) and not necessarily those of the NHS, the NIHR or the Department of Health and Social care.

## Conflict of Interest

The authors declare that the research was conducted in the absence of any commercial or financial relationships that could be construed as a potential conflict of interest.
