# Supplementary material for: Attenuated Induction of the Unfolded Protein Response in Adult Human Primary Astrocytes in Response to Recurrent Low Glucose
Source: Front Endocrinol (Lausanne). 2021 May 26;12:671724. doi: 10.3389/fendo.2021.671724 (PMC8187939; doi:10.3389/fendo.2021.671724)
Supplement: Supplementary file 2 [file DataSheet_2.docx]

## Astrocyte isolation and cell culture.

HPA cells were isolated from normal subventricular deep white matter blocks immediately post-mortem following consent from next-of-kin and with ethical approval from the North and East Devon Research Ethics Committee as previously described [5]. The recurrent low glucose (RLG) model has been previously described [6]. Each day cells were cultured in 2.5 mmol/L glucose-containing media for 2 hours before being changed for media containing 0.1 (low) or 2.5 (normal) mmol/L glucose for 3 hours. To control for differences in osmolarity, 22.5 or 24.9 mmol/l mannitol was added to media containing 2.5 or 0.1 mmol/L glucose respectively. Overnight, cells were recovered in stock media containing 5.5 mmol/L glucose. This was repeated for four days. Control and low glucose (LG) treated cells had 2.5 mmol/L glucose for three days and on the fourth day the LG group received low glucose. The antecedent RLG (aRLG) and RLG groups had 0.1 mmol/L glucose for fourth days, except on the fourth day the aRLG groups had 2.5 mmol/L glucose. Samples were split for RNA extraction and DNA extraction, with a total of five and six replicates for RNA sequencing and DNA methylation studies, respectively. Cells were confirmed as mycoplasma free using the MycoAlert kit (Lonza, Slough, UK).

## RNA sequencing

Briefly, RNA was extracted using TRIzol and Direct-zol miniprep kit (Invitrogen, Carlsbad, CA, USA), according to manufacturers’ instructions. cDNA libraries were generated using the TruSeq DNA HT Library Preparation Kit (Illumina Inc., San Diego, CA, USA). Sequencing reads were generated using the Illumina HiSeq 2500 and fastq sequence quality was checked using MultiQC before alignment to the human genome (Build GRCh38.p12) using STAR. Mapped reads were counted using the FeatureCounts function of the subread package. Differential gene expression was calculated using DESeq2 using the Likelihood ratio test function to analyse all groups together followed by the Wald-test for pairwise analysis. Genes with a false discovery rate (FDR) ≤0.05 were considered differentially expressed. Functional gene ontology analysis was performed using GOSeq. Gene length was accounted for during GO analysis. Raw RNAseq files are available at GEOLINK.

## DNA methylation analysis

DNA was extracted using a modified phenol:chloroform protocol. DNA methylation was measured using the Infinium MethylationEPIC BeadChip platform (Illumina Inc.) (EPIC). 729727 probes remained after QC processes. The one-way analysis of variance (ANOVA) test was used to test for differentially methylated sites associated across the three groups: LG, aRLG, RLG compared to controls. To determine which group was driving the association behind the significant ANOVA results, the *T* statistics for controls versus each of the three groups were extracted from the regression model. Power was calculated using <https://epigenetics.essex.ac.uk/shiny/EPICDNAmPowerCalcs/>.
